# Elevation of Global *O*-GlcNAc in Rodents Using a Selective *O*-GlcNAcase Inhibitor Does Not Cause Insulin Resistance or Perturb Glucohomeostasis

**DOI:** 10.1016/j.chembiol.2010.07.005

**Published:** 2010-09-24

**Authors:** Matthew S. Macauley, Xiaoyang Shan, Scott A. Yuzwa, Tracey M. Gloster, David J. Vocadlo

**Affiliations:** 1 Department of Chemistry, Simon Fraser University, Burnaby, BC V5A 1S6, Canada; 2 Department of Molecular Biology and Biochemistry, Simon Fraser University, Burnaby, BC V5A 1S6, Canada

## Abstract

The *O*-GlcNAc modification is proposed to be a nutrient sensor with studies suggesting that global increases in *O*-GlcNAc levels cause insulin resistance and impaired glucohomeostasis. We address this hypothesis by using a potent and selective inhibitor of *O*-GlcNAcase, known as NButGT, in a series of in vivo studies. Treatment of rats and mice with NButGT, for various time regimens and doses, dramatically increases *O*-GlcNAc levels throughout all tissues but does not perturb insulin sensitivity or alter glucohomeostasis. NButGT also does not affect the severity or onset of insulin resistance induced by a high-fat diet. These results suggest that pharmacological increases in global *O*-GlcNAc levels do not cause insulin resistance nor do they appear to disrupt glucohomeostasis. Therefore, the protective benefits of elevated *O*-GlcNAc levels may be achieved without deleteriously affecting glucohomeostasis.

## Graphical abstract

**Graphical Abstract:**

**Figure:**
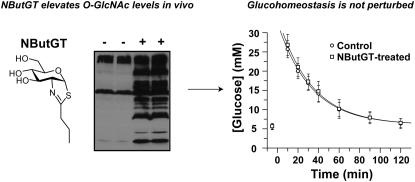

**Highlights:**

► Oral or intravenous delivery of NButGT increases *O*-GlcNAc levels in all tissues assayed ► Various rodent models of insulin resistance were studied using NButGT ► Inhibition of OGA does not perturb insulin sensitivity or alter glucohomeostasis ► Provides framework for future studies examining the *O*-GlcNAc modification in vivo

## Introduction

Insulin activates signaling pathways that maintain glucohomeostasis in many tissues but most importantly in muscle, liver, and adipose tissue. In recent years, there has been a growing appreciation that activation of these signaling pathways can be fine-tuned by additional mechanisms. Nutrient availability has been one focus of research in this area, with numerous studies demonstrating a link between nutrient availability and insulin sensitivity (Liu et al., 2008; Schenk et al., 2008; Towler and Hardie, 2007; Wang and Proud, 2009). Hyperglycemia has long been known to cause insulin desensitization (Kim et al., 2001; Nawano et al., 2000; Nelson et al., 2002; van Putten and Krans, 1985). The effects of chronically elevated blood glucose levels on various tissues clearly play an important role in the self-amplifying nature of type II diabetes mellitus; however, the molecular mechanism(s) by which glucose influences insulin sensitivity is not fully understood and continues to be a topic of considerable interest.

A small percentage of glucose enters the hexosamine biosynthetic pathway (HBSP) to make UDP-GlcNAc, which is in turn used for the biosynthesis of UDP-GalNAc and CMP-NeuAc, and together these three molecules are used by various cellular glycosyltransferases for the biosynthesis of many different glycoconjugates. Numerous studies have shown that increased cellular UDP-GlcNAc levels correlate with insulin resistance (Hebert et al., 1996; Marshall et al., 1991; Rossetti et al., 1995). One form of glycosylation that depends exclusively on UDP-GlcNAc is the intracellular glycosylation of nucleocytoplasmic proteins with a single β-*O*-linked *N*-acetylglucosamine (GlcNAc) residue. Levels of *O*-GlcNAc on proteins are regulated by two enzymes: *O*-GlcNAc transferase (OGT) catalyzes the installation of GlcNAc onto specific serine and threonine residues of target proteins via a β-glycosidic linkage, while *O*-GlcNAcase (OGA) removes the modification. A leading hypothesis for the cellular role(s) of the *O*-GlcNAc modification is that an interplay between *O*-GlcNAc and phosphorylation exists to fine-tune cellular signaling pathways. As the *O*-GlcNAc modification has been detected on a number of proteins involved in the insulin signaling cascade, such as Akt (Park et al., 2005), IRS-1 (Ball et al., 2006), and FoxO1 (Housley et al., 2008), elevated *O*-GlcNAc levels have the potential to be a molecular mechanism that links hyperglycemia to insulin resistance.

Based on a number of in vivo studies, elevation of cellular *O*-GlcNAc levels have been proposed to cause insulin resistance or perturb glucohomeostasis. The most compelling of these studies observed insulin resistance resulting from adenoviral-mediated overexpression of OGT in the liver (Dentin et al., 2008; Yang et al., 2008) or by transgenic overexpression of OGT in muscle and fat tissue (McClain et al., 2002). As discussed in Macauley et al. (2010) (this issue of *Chemistry & Biology*), increasing *O*-GlcNAc levels in a classical adipocyte model using two structurally distinct OGA inhibitors does not support a role for elevated *O*-GlcNAc playing an independent role in the development of insulin resistance. Therefore, the role that increased *O*-GlcNAc levels play in the development of insulin resistance in cultured cells is controversial and investigating the role of *O*-GlcNAc in the context of an intact endocrine system might provide a more faithful environment in which to discern effects arising from increased *O*-GlcNAc levels. Accordingly, we felt that exploring the effects of increased *O*-GlcNAc levels induced by the inhibition of OGA in vivo would be an important contribution. The use of OGA inhibitors would have the advantage that they could aid understanding of the temporal relationship between cellular *O*-GlcNAc levels and insulin resistance as well as to evaluate if this process is reversible. On the other hand, if no insulin resistance or other disruptions in glucohomeostasis are observed this would indicate *O*-GlcNAc levels, stemming from inhibition of OGA, are not an independent mechanism leading to insulin resistance. Such a finding would be notable since it would open the door to detailed study on the independent physiological functions of increased *O*-GlcNAc levels. Because in Macauley et al. (2010), we find that two structurally distinct inhibitors both have similar effects on *O*-GlcNAc levels and signaling pathways, we initiated studies in rodents using the selective OGA inhibitor known as NButGT.

Here, we find that NButGT elevates *O*-GlcNAc levels throughout all tissues tested of Sprague-Dawley rats and C57BL/6J mice but, surprisingly, animals treated for a prolonged period of time with high doses of NButGT are healthy and show no signs of insulin resistance as determined by glucose tolerance tests and hyperinsulemic euglycemic clamp. Therefore, studies in cultured cells and in vivo appear to be in agreement that selective inhibition of OGA does not cause insulin resistance. Nevertheless, these findings are not in harmony with studies overexpressing OGT (Dentin et al., 2008; McClain et al., 2002; Yang et al., 2008). A discussion is presented on some factors that might account for this discrepancy between pharmacological and genetic methods used to increase *O*-GlcNAc levels. The findings of this study open the intriguing possibility of elevating *O*-GlcNAc levels throughout an entire organism, by using a small molecule inhibitor of OGA, not only for advancing our understanding of the in vivo roles of the *O*-GlcNAc modification, but also for beneficial purposes such as exploiting the protective effect that elevated *O*-GlcNAc levels have been shown to confer to cells and tissues (Ngoh et al., 2008; Zachara et al., 2004; Zou et al., 2007).

## Results and Discussion

The effect of elevated *O*-GlcNAc levels, induced by a small molecule inhibitor of OGA, on glucohomeostasis in vivo has never been investigated. We felt that exploring the effects of the OGA inhibitor NButGT in vivo, where the entire endocrine system is operational, would be a valuable study complimenting existing reports in which *O*-GlcNAc levels have been modulated using genetic approaches (Dentin et al., 2008; McClain et al., 2002; Yang et al., 2008). Two recent studies have suggested that such a study might be feasible; two different small molecule OGA inhibitors, PUGNAc and Thiamet-G, have been shown to increase global *O*-GlcNAc levels in rodents (Yuzwa et al., 2008; Zou et al., 2007). We felt that NButGT would be an excellent candidate for these studies since (1) it would enable us to compare our results with previous data using cultured adipocytes (Macauley et al., 2008), (2) NButGT is highly selective for OGA (Macauley et al., 2005), (3) it can be readily synthesized on a large multigram scale, and (4) another structurally distinct inhibitor described in Macauley et al. (2010) reveals the same effects on cells as NButGT. Conversely, we felt that PUGNAc would be a poor choice to use in vivo since it does not penetrate all tissues (Zou et al., 2007), inhibits the functionally related HexA and HexB enzymes whose dysfunction leads to lysosomal storage disorders (Triggs-Raine et al., 2001), and results in responses that differ from two other classes of OGA inhibitor in cultured cells, and until very recently (Goddard-Borger and Stubbs, 2010) access to significant quantities of PUGNAc was not feasible. On the basis of these careful considerations, we undertook such studies using NButGT in rodents.

### Administration of NButGT Elevates O-GlcNAc Levels in Sprague-Dawley Rats

**Figure 1:**
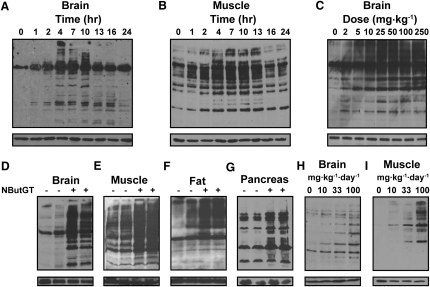
NButGT Globally Elevates *O*-GlcNAc Levels in Sprague-Dawley Rats through Intravenous or Oral Delivery (A and B) Nine rats were treated with NButGT via a tail vein injection at a dose of 50 mg·kg^-1^ and sacrificed at various times postinjection and analyzed for *O*-GlcNAc content in the brain (A) and muscle (B) tissue. (C) Sprague-Dawley rats were given varying doses of NButGT via a tail vein injection and 7 hr after delivery of the inhibitor, the animals were sacrificed, tissues were collected, and *O*-GlcNAc content in the brain was analyzed. (D–G) Two rats were treated with either 0 or 100 mg·kg^-1^·day^-1^ of NButGT incorporated into their chow for 3 days and analyzed for *O*-GlcNAc content in the brain (D), muscle (E), fat (F), and pancreas (G) tissues. (H and I) NButGT elevates *O*-GlcNAc levels in brain (H) and muscle (I) in a dose-dependent manner when incorporated into the chow and fed for 3 days. For all blots, the upper panel represents blots probed with the CTD110.6 anti-*O*-GlcNAc antibody and the lower panel represents blots probed with anti-β-actin as a loading control. For additional experiments supporting data from this figure, see Figure S1.

Preliminary experiments were carried out to assess the effectiveness of NButGT in SD rats. We were particularly interested in the time-dependent effect of NButGT on *O*-GlcNAc levels since longer term studies would require a carefully considered dosing regimen to maintain increased *O*-GlcNAc levels through the entire course of the study. To investigate this issue, SD rats were given an intravenous injection of NButGT at a concentration of 50 mg·kg^-1^ and sacrificed at various times postinjection. Evaluation of homogenized brain and muscle tissues by western blot analyses using the CTD110.6 anti-*O*-GlcNAc antibody (Comer et al., 2001) reveals that *O*-GlcNAc levels are increased in a time-dependent manner in response to NButGT administration (Figures 1A and 1B). Increased *O*-GlcNAc levels are observed after 1 hr and return to approximately basal levels after 24 hr. A study of the dose-dependent increases in *O*-GlcNAc levels in brain revealed that 5 mg·kg^-1^ of NButGT increases *O*-GlcNAc levels (Figure 1C). Pharmacokinetic evaluation of NButGT in blood reveals that NButGT clearance follows an exponential decay with a half-life of 30 min (see Figure S1 available online). Therefore, it appears that clearance of NButGT from the blood is rapid. To maintain increased *O*-GlcNAc levels throughout a 24 hr period, we reasoned that a slow continuous dose of NButGT would be preferable; therefore, we investigated the effect of NButGT incorporated into the animal chow. A 3 day oral treatment using 100 mg·kg^-1^·day^-1^ of NButGT, incorporated into chow, produced elevated levels of *O*-GlcNAc-modified proteins in muscle, fat, brain, and pancreas (Figures 1D–1G), as well as liver and spleen tissues (Figure S1). In a separate study, the dose of NButGT in the food was varied and, based on analyses of brain and muscle tissue, it appears that the oral dose-dependency of *O*-GlcNAc levels were approximately linear up to the largest dose tested of 100 mg·kg^-1^·day^-1^ (Figures 1H and 1I). Collectively, these results show that intravenous or oral administration of NButGT acts in vivo throughout all tissues tested to elevate *O*-GlcNAc levels in both a dose- and time-dependent manner.

### Acute Treatment of Rats with NButGT Does Not Alter Glucose Tolerance

**Figure 2:**
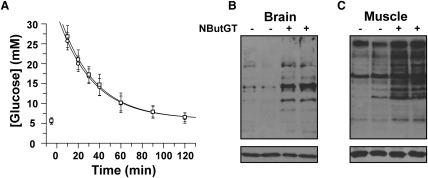
An Acute Treatment with NButGT Does Not Perturb Glucose Tolerance in SD Rats (A) Food was withheld from animals overnight and animals were given a 50 mg·kg^-1^ injection of NButGT (circles) or vehicle (squares) (n = 4). The following morning, 16 hr later, another injection of NButGT or vehicle was given. Seven hours later, a time at which increases in levels of *O*-GlcNAc-modified proteins are maximal, an IVGTT was performed using a 1 g·kg^-1^ bolus IV injection of glucose (t = 0). (B and C) Western blot analyses of homogenized brain (B) and muscle (C) tissue reveal that animals treated with NButGT (+) had elevated levels of *O*-GlcNAc-modified proteins compared with control animals (−) (upper panel). Equivalent protein loading is demonstrated using an anti-β-actin antibody (lower panel).

We next turned our attention to what effect NButGT might have on glucohomeostasis. Six-week-old male SD rats were given a 50 mg·kg^-1^ tail vein injection of NButGT or vehicle and, after 7 hr, we carried out intravenous glucose tolerance tests (IVGTT). Glucose tolerance was unaffected (Figure 2A) even though animals treated with the inhibitor had dramatic increases in levels of *O*-GlcNAc-modified proteins (Figures 2B and 2C). Additionally, resting blood glucose levels, which are a classic measure of insulin sensitivity and reflective of the severity of insulin resistance (Muniyappa et al., 2008), were unaffected by inhibitor treatment and the resulting increased global *O*-GlcNAc levels (Figure 2A). This finding differs somewhat from the findings of Dentin et al. (2008) which showed that adenoviral-mediated overexpression of OGT in liver caused elevated blood glucose levels in animals fed ad libitum. Also differing from our findings is that Yang et al. (2008) reported that when OGT was overexpressed in the liver of mice, through adenoviral-mediated infection, insulin sensitivity was diminished but elevated blood insulin levels masked any effect on resting blood glucose levels and glucose tolerance. In both these studies, the approach to increasing *O*-GlcNAc levels was quite different in that genetic methods were used to overexpress OGT in a targeted tissue. Considering this difference and the possibility that insulin resistance may take more time to develop, we therefore undertook studies in which insulin levels were monitored, animals were dosed for longer times, and more rigorous measures of glucohomeostasis were used.

### NButGT Does Not Perturb Activation of the Insulin Signaling Pathway in Liver and Muscle

**Figure 3:**
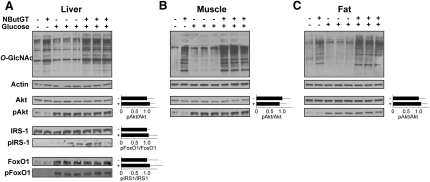
NButGT Does Not Perturb Activation of Key Insulin Signaling Molecules (A–C) Four SD rats were treated for 3 days with 1000 mg·kg^-1^·day^-1^ of NButGT. Four animals were also treated as controls. On the fourth day, the animals were given either a tail vein injection of 1 g·kg^-1^ of glucose or PBS as a control. Precisely 10 min later, the animals were sacrificed and the liver, muscle, and adipose tissues were harvested for western blot analyses. Western blots from homogenized liver (A), muscle (B), and adipose (C) tissue were probed with the indicated antibodies. Densitometry of the relevant blots is shown directly to the right; in no cases does NButGT cause a statistically significant difference in the insulin-mediated phosphorylation of these signaling molecules.

The results presented thus far have all probed functional measures of insulin resistance. Previous results using overexpression of OGT or PUGNAc have suggested that increased *O*-GlcNAc levels prevent phosphorylation of key signaling molecules in the insulin signaling cascade such as IRS-1 and Akt (Dentin et al., 2008; Yang et al., 2008). To look for insulin resistance at the molecular level, SD rats were treated orally for 3 days with 1000 mg·kg^-1^·day^-1^ of NButGT. On the third day, the food was withdrawn overnight and NButGT was placed in the water at the same dose in order maintain *O*-GlcNAc levels. The following day, animals were given a 1 g·kg^-1^ tail vein injection of glucose or PBS as a control. After 10 min, the animals were sacrificed and the appropriate tissues were collected for analysis by western blotting. NButGT treatment, and the associated increases in *O*-GlcNAc levels, did not impair insulin-stimulated activation of Akt, IRS1, or FoxO1 in the liver while Akt activation was also not impaired in muscle or fat tissues (Figure 3). These results are in keeping with the functional measures of insulin sensitivity described above as well as our previous report that NButGT does not prevent insulin-stimulated activation of Akt in 3T3-L1 adipocytes (Macauley et al., 2008).

### Two-Week Treatment of Rats with NButGT Does Not Alter Glucose Homeostasis

**Figure 4:**
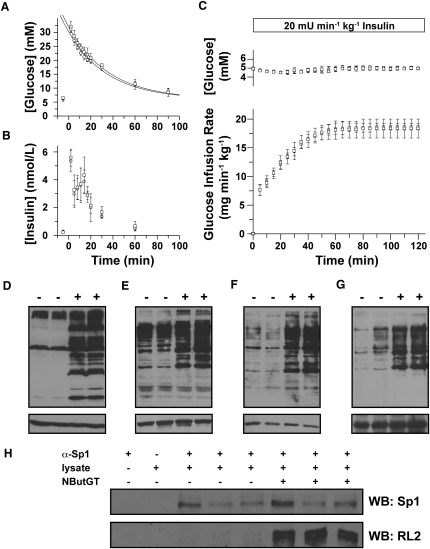
Two Week Treatment of Rats with NButGT Does Not Perturb Glucohomeostasis (A–C) Eight animals were treated orally with NButGT at a dose of 200 mg·kg^-1^·day^-1^ (circles) for 2 weeks while eight control animals were treated identically except that no inhibitor was added to their food (squares). (A) An IVGTT was performed using a 1 g·kg^-1^ bolus IV injection of glucose (t = 0) and blood glucose levels were analyzed as a function of time. (B) Blood insulin levels throughout the glucose challenge were analyzed for insulin content. (C) A separate set of animals (n = 6) were treated identically for 2 weeks and subjected to a hyperinsulemic-euglycemic clamp using an insulin infusion rate of 20 mU·min^-1^·kg^-1^. Data are represented as mean ± SD. Under no conditions are there differences observed between the treated and control groups. (D–G) Rats treated with NButGT for 2 weeks have elevated *O*-GlcNAc levels. Analysis of *O*-GlcNAc levels in brain (D), muscle (E), fat (F), and liver (G) tissue from treated (+) and control (−) animals using CTD110.6 (upper panels). Equivalent protein loading is demonstrated in the lower panels using an anti-β-actin antibody (lower panels). (H) Immunoprecipitation of Sp1 from the spleen of 2 week treated animals. Immunoprecipitates were probed for Sp1 and the RL2 anti-*O*-GlcNAc antibody. For additional experiments supporting data from this figure, see Figure S2.

Eight rats were dosed orally for 2 weeks with NButGT at a dose of 200 mg·kg^-1^·day^-1^, a dose that would maintain *O*-GlcNAc levels throughout a 24 hr period, based on our pharmacokinetic analysis. Eight weight and age-matched animals were treated as controls. On day 14 of the treatment, an IVGTT was performed and the results show that NButGT treatment does not alter the response to the glucose challenge nor does it affect resting blood glucose levels (Figure 4A). Significantly, we observe no differences in plasma insulin levels between control and treated animals before or after the glucose challenge (Figure 4B). To rigorously probe for the presence of insulin resistance in peripheral tissues we repeated the two week treatment study on six experimental and six control animals and carried out hyperinsulemic-euglycemic clamp experiments—the gold standard for measurement of insulin sensitivity of peripheral tissues in vivo (Muniyappa et al., 2008). For this study, 20 mU·min^-1^·kg^-1^ of insulin was infused throughout the 2 hr experiment to ensure that hepatic glucose output was not an interfering variable (Rossetti and Giaccari, 1990). Consistent with the findings of the IVGTT, resting blood glucose levels, and insulin levels, these clamp experiments also did not reveal differences between the insulin sensitivity of peripheral tissues from treated and control groups (Figure 4C).

We had considered that the required fasting of animals overnight prior to the IVGTT and clamp studies might limit the acute exposure of the animals to NButGT prior to testing and thus cause *O*-GlcNAc levels to return to basal levels. To circumvent this problem, NButGT was added to the water supply of the treated animals during the night preceding testing to ensure increased *O*-GlcNAc levels were maintained at the time of the experiments; a prior study with the NButGT analog Thiamet-G had showed that it was orally available when administered through the water supply of the animals (Yuzwa et al., 2008). Following these experiments, tissues from several postclamp animals were analyzed and the levels of *O*-GlcNAc-modified proteins in the brain, muscle, fat, and liver were elevated in NButGT-treated animals (Figures 4D–4G), and these results show that treated animals have dramatically elevated *O*-GlcNAc levels. In addition, immunoprecipitation of Sp1 from the tissues of three control and treated animals shows that *O*-GlcNAc levels are elevated on this protein (Figure 4H).

### Administration of NButGT for 8 Months Does Not Result in Insulin Resistance

**Figure 5:**
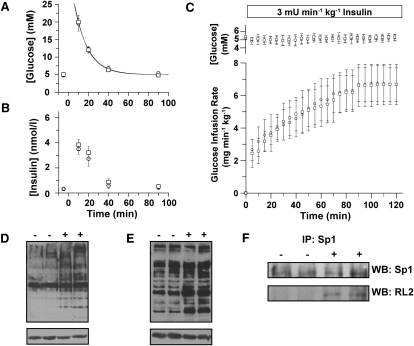
Eight Month Treatment of Rats with NButGT Does Not Perturb Glucohomeostasis (A–C) Six animals were administered NButGT orally at a dose of 200 mg·kg^-1^·day^-1^ (circles). Eight control animals were also treated identically except no inhibitor was added to their food (squares). (A) An IVGTT was performed using a 1 g·kg^-1^ bolus IV injection of glucose (t = 0) and blood glucose levels were analyzed as a function of time. (B) Blood insulin levels throughout the glucose challenge were determined. (C) A week later, the animals were subjected to a hyperinsulemic-euglycemic clamp using an insulin infusion rate of 3 mU·min^-1^·kg^-1^. Data represented as mean ± SD. (D and E) Analysis of *O*-GlcNAc levels in brain (D) and muscle (E) from treated (+) and control (−) animals using CTD110.6 (upper panels). Equivalent protein loading is demonstrated with an anti-β-actin antibody (lower panels). (F) Immunoprecipitation of Sp1 from the spleen of animals treated with (+) and without (−) NButGT for 2 weeks. Immunoprecipitates were probed for Sp1 and RL2.

Given these findings, we considered that the onset of insulin resistance and type II diabetes is typically slow. Chronic elevation of *O*-GlcNAc levels, rather than acute increases, could cause a gradual adaptation within tissues giving rise to a physiologically realistic slow onset of insulin resistance. This hypothesis seems consistent with observations that acute fluctuations in dietary intake cause oscillations in *O*-GlcNAc levels (Akimoto et al., 2001, 2007) and that prolonged excess nutrient intake could cause gradual accumulation of insults via *O*-GlcNAc and thus trigger insulin resistance. To evaluate the effects of chronic elevation of *O*-GlcNAc levels, we carried out a long-term study using six male SD rats treated with 200 mg·kg^-1^·day^-1^ of NButGT and eight control rats. Resting blood glucose levels, food and water consumption, and the body weight of these animals, all of which are measures that can reveal a diabetic phenotype, were monitored closely over the course of the study. Surprisingly, no differences were observed between the two groups for any of these variables (Figure S2). As well, no differences were observed in triglycerides, free fatty acids, and leptin, or in the weights of individual organ weights upon sacrifice (Figure S2). Treated animals did not show any sign of discomfort or any abnormal behavior as monitored during regular handling. Following 8 months of treatment, animals were subjected to an IVGTT and, later, a hyperinsulemic-euglycemic clamp. We observed no differences between groups with respect to glucose tolerance, insulin secretion during IVGTT, or insulin sensitivity (Figures 5A–5C). The advanced age of these animals and their natural variation in weight gave rise to expected levels of variation in the clamp results (James et al., 1985).

For this study, the inhibitor was not delivered in the water during the overnight fast since the aim was to evaluate differences stemming from chronic elevation of *O*-GlcNAc levels and not the potential acute effects of increased *O*-GlcNAc levels. Despite this overnight fasting and consequent 19 hr withdrawal of inhibitor, significant increases in the levels of *O*-GlcNAc-modified proteins were still observed in brain and muscle (Figures 5D and 5E), which is in keeping with our pharmacokinetic results. As well, immunoprecipitation of Sp1 revealed that its *O*-GlcNAc levels were elevated by inhibitor treatment (Figure 5F). Notably, these differences in *O*-GlcNAc levels are observed despite an approximately 3-fold increase in OGA levels in treated versus control animals while no significant change in OGT levels were observed (Figure S2). These findings indicate that pharmacological interventions maintain increased *O*-GlcNAc levels over the long term, even with the elevation in OGA levels observed in treated animals. The increases in *O*-GlcNAc levels observed in the long-term study, even after 19 hr withdrawal of the inhibitor, are as large as those seen under hyperglycemic conditions (Akimoto et al., 2001; Walgren et al., 2003) or in diabetic animals (Akimoto et al., 2007).

### Prolonged Global Increases in *O*-GlcNAc Levels Do Not Cause β-Cell Toxicity

Alterations in *O*-GlcNAc levels have also been proposed to mediate impaired glucose-stimulated insulin secretion (GSIS) since elevated *O*-GlcNAc levels have been correlated with decreased GSIS (Akimoto et al., 2007; Gao et al., 2000; Liu et al., 2000). Other studies, however, do not support this hypothesis (Kaneto et al., 2001; Zraika et al., 2006). A further report showed that elevating *O*-GlcNAc levels resulted in increased insulin production rather than impaired insulin secretion (Andrali et al., 2007). It is difficult to reconcile these findings, which appear to suggest opposing effects, but it is notable that several of these studies have used enzyme inhibitors with established off-target effects such as PUGNAc and streptozotocin. In an effort to clarify these conflicting observations, we examined the long-term treated animals for any signs of β cell toxicity. Following the 8 month study, the pancreas of several treated and control animals were analyzed by immunohistochemistry (IHC) (Figure S2). Consistent with the western blot analyses from the brain and muscle tissues of the long-term treated animals, higher *O*-GlcNAc levels were observed in the pancreas of NButGT-treated animals. Qualitative IHC analysis using an anti-insulin antibody and hematoxylin staining of many pancreatic sections revealed no apparent differences in the morphology or number of islets between the groups (Figure S2). These findings are in keeping with our results showing that global increases of *O*-GlcNAc levels do not give rise to differences in fasting blood insulin levels nor do they alter insulin secretion in response to glucose challenge (Figures 4B and 5B).

### High Dose of NButGT for 2 Months Does Not Perturb Glucohomeostasis or Elevate the Ganglioside GM2

**Figure 6:**
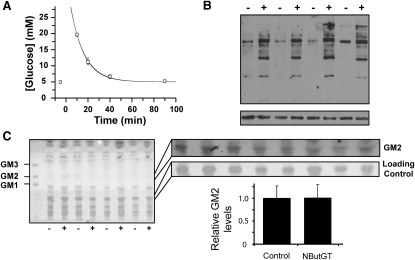
Two Month High-Dose (1000 m g·kg^-1^·day^-1^) Treatment of Rats with NButGT Does Not Perturb Glucose Tolerance or GM2 Levels (A) An IVGTT was performed on four treated (circles) and four control (squares) animals using a 1 g·kg^-1^ bolus IV injection of glucose (t = 0) and blood glucose levels were analyzed as a function of time. (B) Analysis of *O*-GlcNAc levels in the brain of treated (+) and control (−) animals using the CTD110.6 anti-*O*-GlcNAc antibody (upper panel). Equivalent protein loading is demonstrated with an anti-β-actin antibody (lower panel). (C) Analysis of the ganglioside GM2 levels in the brain of treated (+) and control (−) animals. The TLC plate was stained with resorcinol and represents total gangliosides from the brain. The right panels represent a magnified image of the band corresponding to GM2 and another band as a loading control. The panel for GM2 was contrasted for display and densitometry. Densitometric analysis of the GM2 band relative to the load control band reveals no statistical difference in levels of GM2 levels between the two groups.

To rule out the possibility that the dose of NButGT used in the previous studies (200 mg·kg^-1^·day^-1^) was not high enough to observe an effect on glucohomeostasis, four SD rats were treated with a high dose (1000 mg·kg^-1^·day^-1^) of NButGT. After 2 months of treatment, these animals, along with the appropriately treated control animals, were subjected to an IVGTT. Once again, no difference in resting blood glucose levels or glucose tolerance was observed (Figure 6A). In this study, for the overnight fasting period the inhibitor was placed in the water of the treated animals at the same dose and this did give rise to dramatically elevated *O*-GlcNAc levels (Figure 6B).

An additional aim of this particular study was to determine if high levels of NButGT might accumulate and inhibit functionally related enzymes of OGA. In addition to OGA, mammals have several other functionally related enzymes that both cleave terminal β-linked *N*-acetylglucosamine residues and use the same enzymatic mechanism (Macauley and Vocadlo, 2009). HexA and HexB are isozymes that reside in the lysosome and cleave GlcNAc and GalNAc from a variety of glyconjugates. The best characterized substrate for these lysosomal β-hexosaminidases is the ganglioside GM2. Mutations of these enzymes in humans (Triggs-Raine et al., 2001) or deletion of either of the enzymes in mice (Sango et al., 1995; Yamanaka et al., 1994) results in a slow accumulation of GM2 in the lysosome eventually leading to lysosomal storage disorders known as Tay-Sachs and Sandhoff disease and a dramatically shortened life span. The most commonly used inhibitor of OGA, PUGNAc, suffers from the shortcoming that it is an equally potent inhibitor of both OGA and the lysosomal β-hexosaminidases. This nonspecific effect results in both elevated *O*-GlcNAc levels and GM2 levels in cultured neuroblastoma cells (Stubbs et al., 2009) as well as BV2 microglia cells (Ho et al., 2010). In contrast, NButGT was designed to deliver selectivity for OGA over the lysosomal β-hexosaminidases (Macauley et al., 2005). Indeed, the 600-fold selectivity of NButGT for OGA has been shown to prevent it from increasing GM2 levels in cultured cells (Stubbs et al., 2009). To evaluate whether NButGT acts to elevate GM2 levels over prolonged dosing periods, gangliosides were extracted from brain tissue of animals in the 2 month high-dosing study. Although gangliosides are found in all tissues, the brain is particularly rich in gangliosides, making it a valuable reporter for ganglioside levels. As shown in Figure 6C, NButGT-treated SD rats did not exhibit increased GM2 levels in their brains. In comparison, *Hexb^−/−^* mice show dramatic accumulation of GM2 levels to levels at least 10-fold above healthy animals at 2 months of age (Andersson et al., 2004). These results underscore the selectivity of NButGT over the functionally related HexA and HexB and provide good evidence that this selectivity extends to in vivo models even at very high doses.

### NButGT Does Not Perturb Glucose Homeostasis in Mice or Affect the Onset or Severity of Insulin Resistance Induced by a High-Fat Diet

**Figure 7:**
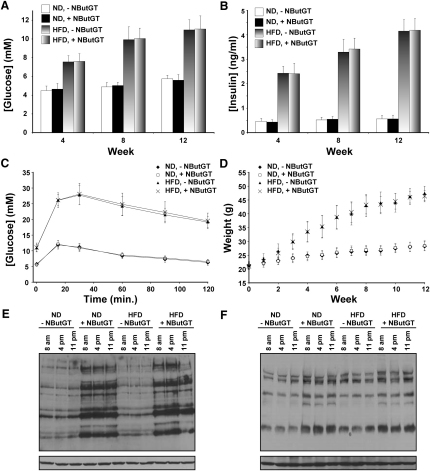
Three Month Treatment of Mice with 1000 mg·kg^-1^·day^-1^ NButGT Does Not Perturb Glucohomeostasis (A) Resting blood glucose levels of treated and control animals on a normal diet (ND) or high-fat diet (HFD) at 4, 8, and 12 weeks after starting dosing (n = 12). (B) Resting blood insulin levels at 4, 8, and 12 weeks after starting dosing (n = 12). (C) An oral glucose tolerance test (OGTT) was performed after 13 weeks of treatment (n = 12 per group). (D) Growth rate of mice on a normal diet (ND) or high-fat diet (HFD). Body weight was recorded once a week over the course of the study (n = 12 per group). (E and F) 1000 mg·kg^-1^·day^-1^ of NButGT maintains elevated *O*-GlcNAc levels throughout a 24 hr cycle in the muscle (E) and liver (F) of mice on a normal diet (ND) or high-fat diet (HFD). One animal from all four groups was sacrificed at 8 a.m., 4 p.m., and 11 p.m. and analyzed by western blot for *O*-GlcNAc levels (upper panel) and for protein loading using an anti-β-actin antibody (lower panel).

All experiments described thus far were carried out in SD rats; however, in some experimental paradigms, variance within rodents is observed. For instance, a percentage of SD rats are resistant to weight gain when placed on a high-fat diet (Levin et al., 1987). To evaluate whether the absence of any observable effect on insulin resistance that we observe when treating SD rats with NButGT is a general phenomenon in rodents, we tested NButGT in C57BL/6J mice. As the metabolism of mice is faster than rats and generally leads to faster clearance of small molecules (Hayes, 2007), we used the higher dose of NButGT (1000 mg·kg^-1^·day^-1^) that was used in the experiments involving SD rats. Twelve mice were treated with NButGT for 3 months by incorporating this inhibitor into their chow to provide a dose of 1000 mg·kg^-1^·day^-1^. Resting blood glucose and insulin levels were measured at 4, 8, and 12 weeks after commencement of dosing. At no point was any difference observed in either of these parameters between treated and untreated mice (Figures 7A and 7B). Following 12 weeks, an oral glucose tolerance test (OGTT) was carried out using gavage of 1 g·kg^-1^ of glucose. We find that glucose clearance was unaffected by treatment with NButGT despite clear increases in global *O*-GlcNAc levels (Figure 7C).

The studies presented thus far present strong evidence that increased global *O*-GlcNAc levels in vivo, induced by NButGT, do not on their own cause insulin resistance. Nevertheless, it remained a distinct possibility that elevated *O*-GlcNAc levels may play a role in exacerbating the speed of onset and/or the severity of insulin resistance when other pathways are malfunctioning. A common method for inducing insulin resistance in vivo is through diet-induced obesity (DIO) using a high-fat diet (HFD). We therefore used a HFD in conjugation with NButGT, to evaluate whether dysfunction of other pathways is required to observe an effect from increased *O*-GlcNAc levels on glucohomeostasis. This study was carried out in parallel with, and using the same dosing regimen, as the previous study using healthy mice. As shown in Figure 7A, the HFD induced a gradual elevation in resting blood glucose levels. The onset or severity of insulin resistance, judged in this manner, was not affected by treatment with NButGT. The resting blood glucose levels that we observe for mice placed on a HFD for 12 weeks closely matches values observed by others for an equivalent length of treatment (Ohtsubo et al., 2005). The resting blood insulin levels also remained unaffected by inhibitor treatment throughout the course of the study, with a 9-fold increase occurring after 12 weeks in HFD mice as compared with healthy mice. An OGTT was carried out and revealed that mice on a high-fat diet cleared glucose slower (Figure 7C) when compared with control animals; however, there was no difference in glucose clearance arising from NButGT treatment and consequent elevation of *O*-GlcNAc. Weight gain in both healthy and HFD mice was not affected by NButGT (Figure 7D). In order to clarify whether the mode of dosing in this study produced increased *O*-GlcNAc levels throughout a 24 hr period, an animal from each control and treated group were sacrificed at three different time points throughout the day (8 a.m., 4 p.m., and 11 p.m.). In both muscle and liver tissues, *O*-GlcNAc levels were elevated in the treated animals at all three time points (Figures 7E and 7F), supporting this oral dosing regimen as a means of generating sustained increases in *O*-GlcNAc levels.

Overall, the results of the studies we describe here differ with previous findings in which genetic methods have been used to overexpress OGT. Those studies have proposed that increased *O*-GlcNAc levels induce insulin resistance and/or disrupt glucohomeostasis (Dentin et al., 2008; McClain et al., 2002; Yang et al., 2008). There are obvious differences between using small molecules to increase *O*-GlcNAc levels and using genetic methods (Knight and Shokat, 2007) and a consideration of these differences is prudent. Various possible scenarios as to why genetic and chemical methods of altering *O*-GlcNAc levels give rise to different effects in vivo include: (1) increases in global *O*-GlcNAc levels may result in increased hepatic output and therefore mask certain physiological measurements, (2) the cycling rate of *O*-GlcNAc on various proteins may play a role in nutrient sensing, (3) NButGT may not gain access to all compartments and may therefore not affect *O*-GlcNAc levels on nutrient sensing proteins, and (4) OGT may have noncatalytic protein scaffolding roles that are independent of increased *O*-GlcNAc levels. A discussion of the likelihood of each of these possible scenarios is presented in the Supplemental Information. These considerations should serve to guide future experiments that may provide insight into the biological roles of *O*-GlcNAc and/or OGT.

## Significance

**We describe the first studies to our knowledge in which global *O*-GlcNAc levels are increased throughout a whole organism for an extended period of time using an OGA inhibitor in order to evaluate the effects on glucohomeostasis. To realize this aim, we have used NButGT, an inhibitor of OGA that is selective for this enzyme, so as to avoid a complex phenotype stemming from concomitant inhibition of functionally related enzymes. We investigated multiple time regimens ranging from 1 day to 8 months of treatment in mice and rats. No signs of functional insulin resistance or perturbed glucohomeostasis were observed as assessed by resting blood glucose and insulin levels, hyperinsulemic-euglycemic clamps, and glucose tolerance tests. Consistent with the absence of any observable effect on functional measures of insulin sensitivity, no signs of insulin resistance at the molecular level are observed from NButGT treatment. The findings described here differ somewhat from previous studies in which OGT has been overexpressed in vivo. The collective observations, including those described here, however offer several interesting hypotheses that could be reconciled and lead to greater clarity in the area of *O*-GlcNAc. Notably, the results here are intriguing in the context of the proposed beneficial effects of increased *O*-GlcNAc levels on survival of cells and tissues faced with various stresses (****Ngoh et al., 2008; Zachara et al., 2004; Zou et al., 2007****). Indeed, the studies we describe here open the possibility that increased *O*-GlcNAc levels, induced using inhibitors of OGA, could be harnessed for therapeutic benefit without disruption of glucohomeostasis. Perhaps most significantly, this study offers guidance for future in vivo studies designed to further our understanding of this unique form of glycosylation.**

## Experimental Procedures

### General

All solutions containing salts and buffers were made from materials obtained from Bioshop unless otherwise noted. The CTD110.6 anti-*O*-GlcNAc, anti-β-actin, and anti-OGT antibodies were purchased from Covance, Sigma, and Santa Cruz, respectively. All secondary antibodies were obtained from Santa Cruz.

### Animal Care

All experiments carried out on animals were approved by the university animal care committee of Simon Fraser University. For studies with rats, 5-week-old Sprague-Dawley rats (Charles River) were acclimatized for 1 week prior to experimentation. For studies with mice, C57BL/6J mice (Charles River) were obtained at 3 weeks of age and allowed to acclimatize for 1 week prior to experimentation. Rooms were kept at 22°C and maintained on a constant 12 hr light/12 hr dark cycle. Food and water were replaced every 2–3 days. See Supplemental Experimental Procedures for details on the synthesis of NButGT. Following the completion of experiments, animals were sacrificed by either a 100 mg·kg^-1^ intravenous injection of euthanyl (Bimeda-MTC) or with CO_2_. Tissues were collected immediately after sacrifice, flash frozen in liquid nitrogen, transported on dry ice, and stored at −80°C until required.

### Preparation of Animal Chow

Powdered standard rodent chow (600 g, 5001 Lab Diet) was combined with 360 ml of water containing the appropriate amount of inhibitor. The amount of added inhibitor was based on an average daily food consumption of 25 and 3 g, respectively, for rats and mice. After thorough mixing by hand, the mixture was passed though a pasta maker (Creative Technologies) and dehydrated for 36 hr at 37°C. Food for the control animals was processed in an identical manner. All food was stored at −20°C until it was used. Chow containing 60% energy from fat was obtained from TestDiet (58Y1). To incorporate NButGT into this chow, the chow was warmed in a 37°C oven for 30 min to soften. The appropriate amount of inhibitor was then added and the chow was mixed in a Kitchen Aid mixer for 5 min to ensure equal distribution. The chow was formed into cylinders, hardened at −20°C, and cut into small chunks. Food for control animals was treated identically. All food was stored at −20°C until it was used.

### Intravenous Glucose Tolerance Test

Animals were fasted overnight (16 hr). Prior to initiating the experiment, resting blood glucose levels were measured. To initiate the experiment, a 1 g·kg^-1^ tail injection of glucose (50% in PBS) was delivered via either the tail vein over 30 s or gavage. At various times, a small quantity of whole blood was used to directly measure the concentration of glucose using a glucometer (Accu-chek Advantage). As well, for studies in which insulin was monitored over the course of the experiment, 200 μl of blood was collected from the saphenous vein. These blood samples were stored on ice for 1 hr and then centrifuged at 2000 rpm for 10 min to obtain serum. Samples were aliquoted and stored at −20°C until further processing.

### Tissue Homogenization

Tissues were homogenized by manual grinding frozen tissues into a fine powder followed by homogenization in cell lysis buffer (50 mM NaH_2_PO_4_, 150 mM NaCl, 0.1% SDS, 1% NP-40, 0.25% sodium deoxycholate, protease inhibitor tablet (Roche), 1 mM EDTA, and 1 mM NButGT [pH 7.4]) using a tissue homogenizer (T-18 Ultra-Turrax, Ika). Two hundred milligrams of the ground tissue was dissolved in 2 ml of homogenization buffer. Insoluble cell debris were removed by centrifugation at 17,900 rcf for 20 min and the resulting supernatant was used immediately or stored at −20°C until required.

### Hyperinsulemic-Euglycemic Clamp

Animals were fasted overnight (16 hr). The following morning, animals were anesthetized with a 60 mg·kg^-1^ intraperitoneal injection of sodium pentobarbital (Bimeda-MTC). The tail vein was then catheterized and sodium pentobarbital was delivered at a constant rate of 11 mg·kg^-1^·min^-1^ (in saline containing 20 U/ml heparin) for the remainder of the experiment in a total volume of 2 ml. While unconscious, a catheter was inserted in the femoral artery and then looped back into the femoral vein to create a blood sampling loop that minimized blood loss (Lautt et al., 1998). For 2 hr, insulin (2.5 ml in total) (Eli Lilly) and a 50% solution of glucose were delivered to the animal via the tail vein catheter. Delivery of insulin was initiated 4 min prior to the glucose. Every 5 min, blood glucose levels were determined using a glucometer and the glucose infusion rate was adjusted to maintain a level of 5 mM throughout the experiment.

### General Procedures

Details describing western blotting, immunoprecipitation, immunohistochemistry, serum analysis, large-scale synthesis of NButGT, pharmacokinetic analysis, and analysis of ganglioside levels are provided in the supporting material.

## Supplemental Information

Document S1. Two Figures, Supplemental Discussion, Supplemental Experimental Procedures, and Supplemental References
