# Elevation of Global *O*-GlcNAc in Rodents Using a Selective *O*-GlcNAcase Inhibitor Does Not Cause Insulin Resistance or Perturb Glucohomeostasis

**DOI:** 10.1016/j.chembiol.2010.10.007

**Published:** 2010-10-29

**Authors:** M.S. Macauley, X. Shan, S.A. Yuzwa, T.M. Gloster, D.J. Vocadlo

(Chemistry & Biology *17*, 949–958; September 24, 2010)

The following conflict of interest statement was inadvertently omitted from the final manuscript during the production process:

M.S.M., X.S., S.A.Y., and D.J.V. are eligible to receive royalties from Simon Fraser University. D.J.V. is a founder, shareholder, consultant, and member of the scientific advisory board of Alectos Therapeutics.

*Chemistry & Biology* apologizes for this omission.

