# Supplementary material for: Elevation of Global O-GlcNAc in Rodents Using a Selective O-GlcNAcase Inhibitor Does Not Cause Insulin Resistance or Perturb Glucohomeostasis
Source: Chem Biol. 2010 Sep 24;17(9):949–58. doi: 10.1016/j.chembiol.2010.07.005 (PMC2954292; doi:10.1016/j.chembiol.2010.07.005)
Supplement: Document S1. Two Figures, Supplemental Discussion, Supplemental Experimental Procedures, and Supplemental References [file mmc1.pdf]

Supplemental Information

Elevation of Global *O*-GlcNAc in Rodents Using a Selective *O*-GlcNAcase Inhibitor Does Not Cause Insulin Resistance or Perturb Glucohomeostasis

Matthew S. Macauley, Xiaoyang Shan, Scott A. Yuzwa, Tracey M. Gloster, and David J. Vocadlo

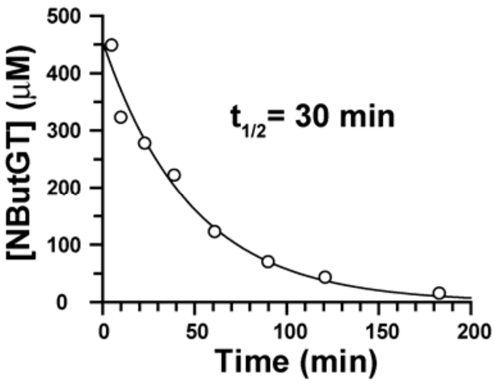

Figure S1, related to Figure 1. Pharmacokinetic analysis of NButGT content in the blood following a 50 mg·kg^{-1} IV injection of the inhibitor
Following delivery of the inhibitor, 200 μL blood samples were taken at various times and spun to collect the plasma. Levels of the inhibitor in the blood were determined by enzyme inhibition assays in conjunction with generation of standard curve for NButGT in the plasma.

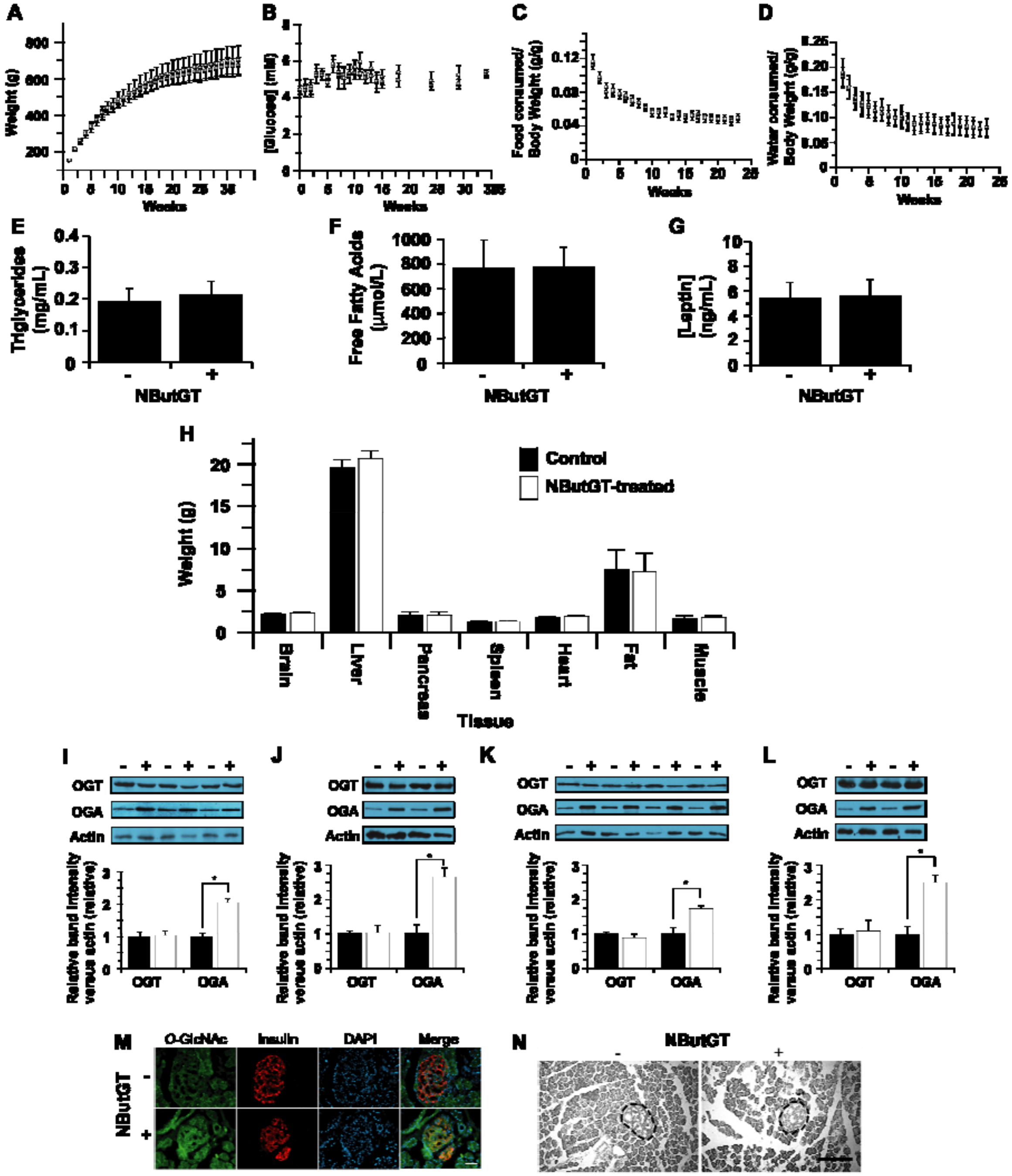

Figure S2, related to Figure 4. Rats treated with NButGT for eight months show no apparent abnormalities in various physiological parameters

(A-D) Six animals were treated orally with NButGT at a dose of 200 $mg \cdot kg^{-1} \cdot day^{-1}$ (circles). Eight control animals were treated identically except no inhibitor was added to their food (squares). Parameters measured were: (A) body weight, (B) resting blood glucose levels, (C) daily food consumption, and (D) daily water consumption as a function of body weight. No differences in any of the parameters are observed between the two groups.

(E-H) Additional statistics from the long-term study of SD rats treated with NButGT does not reveal any differences from prolonged elevations in *O*-GlcNAc-modified proteins.

Levels of (E) triglycerides, (F) free fatty acids, and (G) leptin from the serum of rats treated with NButGT for eight months (+) (n=6) compared to control animals of the same age and weight (-) (n=6). (H) Organs weights from the rats treated with NButGT for eight months (+) (n=6) compared to control animals of the same age and weight (-) (n=6). Error bars are expressed as +/- one standard deviation.
(I,K) Brain and (J,L) muscle tissues were analyzed for levels of OGT and OGA by Western blot analyses from animals treated for two weeks (I,J) or eight months (K,L) with 200 $mg \cdot kg^{-1} \cdot day^{-1}$ NButGT. Densitometry (lower panels) reveal that, in all cases, levels of OGT are not perturbed whereas there is 2-3 fold higher levels of OGA in treated animals (* represents a statistical significance of $p < 0.05$ determined using a t-test).
(M,N) Prolonged increases in *O*-GlcNAc-modified proteins does not perturb β-islet morphology, number or insulin content. (M) Following the long-term study, treated (+) and control (-) animals were perfused and analyzed by immunohistochemistry with anti-*O*-GlcNAc, anti-insulin, DAPI. Data is representative of two animals per group with 18 sections per animal that contained, on average, 12 islets per section. Scale bar: 100 μm at 200x magnification. (N) Pancreatic sections of animals treated with (+) or without (-) NButGT for eight months stained with hematoxylin shows no differences in β-cell morphology. Highlighted area represents an islet. Data is representative of two animals per group and four sections per animal. Scale bar: 200 μm at 100x magnification.

SUPPLEMENTAL DISCUSSION

It is prudent to consider what may account for the apparent differences between this study and previous reports making use of genetic methods to overexpress OGT. Below is a detailed discussion on several scenarios that may account for the different effects between inhibition of OGA and overexpression of OGT on glucohomeostasis. These considerations should serve to guide future experiments that may provide insight into the biological roles of *O*-GlcNAc and/or OGT.

1) Increases in global O-GlcNAc levels result in increased hepatic glucose output.
The primary aim of all of the studies described above was to address the effect of elevated *O*-GlcNAc levels on glucohomeostasis as a whole. The glucose tolerance test and hyperinsulemic-euglycemic clamp are primarily measures of glucose uptake in muscle and fat tissue and so insulin sensitivity in hepatic tissue was not directly measured in these studies. In particular, the hyperinsulemic-euglycemic clamp carried out after the 2-week dosing study used a dose of insulin known to completely suppress HGO (Rossetti and Giaccari, 1990). This was done intentionally to isolate our measurements on the insulin sensitivity of peripheral tissues. On the other hand, the clamp carried out following the long-term 8-month study used a lower dose of insulin. It cannot be concluded, therefore, that HGO was unaffected by NButGT treatment in the long-term study, albeit the lack of difference observed in resting blood glucose levels between groups in any of the experimental paradigms tested does suggest that HGO is unaffected. Further support for a lack of insulin resistance in the liver is that NButGT did not perturb activation of three key signaling molecules (IRS-1, Akt, and FoxO1) in the insulin signaling cascade (Figure 3A). In contrast to the results presented here, decreased insulin sensitivity in the liver and the resulting increased HGO was concluded to be the primary reason for the perturbed glucohomeostasis observed in studies overexpressing OGT in the liver of mice (Dentin et al., 2008; Yang et al., 2008). Experiments carried out in isolated

hepatocytes which compare the effect of OGT overexpression and NButGT on glucose output will be useful to unambiguously clarify this issue.

2) Since O-GlcNAc is a dynamic post-translational modification, the rate at which cycling occurs, rather than the levels of the modification, could be the determinant mediating insulin resistance and disruptions in glucohomeostasis.
Supporting this hypothesis are studies carried in which the genes encoding OGT and OGA are deleted in *C. elegans* (Forsythe et al., 2006; Hanover et al., 2005). These knockouts are viable but do exhibit defects in dauer larva formation. As induction of dauer formation in nematodes is governed by insulin-like signaling pathways (Fielenbach and Antebi, 2008), the authors of these two papers argued that the observed results relate to insulin signaling in mammals. In our studies, cycling should be slowed by treatment with the OGA inhibitor. Interestingly, many studies making use of overexpression of OGT in animals, which results in insulin resistance or perturbed glucose homeostasis, may have increased cycling rates due to the increased levels of OGT. Nevertheless, a clear molecular mechanism that translates cycling rates to changes in cellular physiology has not been proposed. Experiments comparing *O*-GlcNAc cycling rates on proteins of interest in control cells, cells overexpressing OGT, or treated with an OGA inhibitor is an intriguing line of research and may provide insight if this is a possibility for the discrepancy. Accessing this information experimentally, however, will be technically challenging.

3) The use of NButGT may not result in certain key proteins involved in nutrient sensing becoming more heavily modified as may happen during prolonged hyperglycemic conditions or when OGT is overexpressed.
One scenario that could potentially bring about this effect is if OGT had access to a cellular compartment or location where OGA was not present under any circumstances. In this way, overexpression of OGT could increase *O*-GlcNAc levels on certain proteins while an OGA inhibitor would have no effect on levels of *O*-GlcNAc on these proteins. However, the cellular distribution of OGA (Comtesse et al., 2001; Wells et al., 2002; Zeidan et al., 2010) appears to be as broad as OGT (Kreppel et al., 1997; Lubas et al., 1997) and therefore no clear experimental evidence currently supports this scenario. So long as OGA has access to all *O*-GlcNAc-modified proteins, NButGT should elevate *O*-GlcNAc levels globally throughout the cell since competitive inhibition of OGA should not impact the function of OGT, which continues to modify proteins (Macauley and Vocadlo, 2010). Although this possibility cannot be ruled out, we investigated the *O*-GlcNAc modification state of Sp1 (Figures 4H,5F), a key transcription factor regulating expression of multiple genes involved in glucohomeostasis whose *O*-GlcNAc modification state has been suggested to alter its function (Yang et al., 2001). We find that Sp1 glycosylation is significantly increased in both short and long-term studies yet no signs of insulin resistance or disruptions in glucohomeostasis were observed.

4) Overexpression of OGT results in secondary effects that are independent of elevated O-GlcNAc levels.
One concern with using genetic methods is that it remains to be established if, in addition to the catalytic role of OGT, this enzyme also has additional non-catalytic roles. Given that OGT interacts with many protein partners (Cheung and Hart, 2008; Cheung et al., 2008; Fujiki et al., 2009; Housley et al., 2009; Iyer et al., 2003; Marz et al., 2006; Nimura et al., 2009; Slawson et al., 2008) and is found in several large protein complexes (Fujiki et al., 2009; Slawson et al., 2008), overexpression could disrupt and/or introduce protein-protein interactions within cells. Furthermore, OGT has very recently been identified, by

two independent teams, as a member of the polycomb group family (PcG) of proteins (Gambetta et al., 2009; Sinclair et al., 2009), which play a key role in repressing expression of many genes. OGT was also recently shown to activate the histone methyltransferase MLL5 (Fujiki et al., 2009); OGT is therefore an important player in the regulation of gene expression. In light of these observations, the possibility exists that overexpression of OGT within cells may have secondary effects arising from altering expression of enzymes involved in gluconeogenesis, as observed by others (Dentin et al., 2008; Housley et al., 2008; Yang et al., 2008), or by modulation of other cellular processes in which OGT plays an important role through protein-protein interactions. It is notable that Takahashi *et al*. showed that rats treated daily for two years with a high dose of *N*-acetylglucosamine (2.5 $g{\cdot}mg^{-1}{\cdot}day^{-1}$) did not exhibit elevated resting blood glucose levels (Takahashi et al., 2009). As exogenous *N*-acetylglucosamine increases cellular UDP-GlcNAc levels (Boehmelt et al., 2000), and OGT activity has been reported to vary over a broad range of UDP-GlcNAc levels (Kreppel and Hart, 1999), rats dosed with very high doses of *N*-acetylglucosamine are likely to have increased OGT activity and *O*-GlcNAc levels. It is therefore reasonable that overexpression of OGT and inhibition of OGA give rise to different physiological effects as has been observed for other enzyme classes, most notably kinases (Knight and Shokat, 2007). Studies making use of inactive site-directed mutants of OGT (Clarke et al., 2008; Martinez-Fleites et al., 2008) may provide valuable insight into this issue and clarify the role of the OGT protein itself.

SUPPLEMENTAL EXPERIMENTAL PROCEDURES

Synthesis of NButGT

General – Synthetic reactions were monitored by thin layer chromatography (TLC) using Merck Kieselgel 60 F254 aluminum-backed sheets. Compounds were detected on TLC using UV light and charring with 2 M H_2SO_4 in ethanol and heating. Silica gel (230–400 mesh, Merck Kieselgel) flash column chromatography was carried out under a positive pressure with the specified eluants. ^{1}H NMR spectra were recorded on a Varian AS500 Unity Innova spectrometer at 500 MHz.

Chemical Synthesis of NButGT - Large quantities of NButGT were required for these studies (approximately 500 g in total). Optimization of the synthesis for successful large scale preparation of the inhibitor was required. A total of 6 steps is required to prepare NButGT from D-glucosamine, however, a commercially intermediate (1,3,4,6-tetra-*O*-acetyl-2-amino-2-deoxy-β-D-glucopyranose hydrochloride), obtained from CarboSynth, allowed NButGT to be prepared in three steps. The details of the chemical synthesis are described below. NButGT was verified to be >99% pure as determined by ^{1}H NMR spectroscopy and a representative spectra is shown in Figure S2.

Synthesis of 1,3,4,6-tetra-O-acetyl-2-deoxy-2-butyrylamido-β-D-glucopyranose – 1,3,4,6-tetra-*O*-acetyl-2-amino-2-deoxy-β-D-glucopyranose hydrochloride (200 g, 1 eq., 0.516 mol, CarboSynth) was added to 500 mL of DCM and cooled to 4 °C using a large ice bath. Trietheylamine (160 mL, 2.2 eq., 1.13 mol) was added, followed by the slow addition of butyryl chloride (60 mL, 1.1 eq., 0.567 mol). After one hr, a time when the reaction had gone to completion as judged by TLC, the reaction mixture was transferred into a large separatory funnel and washed twice with sodium bicarbonate, twice with water, and once with brine. The organic layer was dried with $MgSO_4$ for two min and then filtered. Following vacuum evaporation of the mixture to one quarter of the original volume, the product was crystallized by the slow addition of hexane. The product was

filtered and dried overnight in the atmosphere on absorbent filter paper in a fume hood. The yield was typically > 90% for this step. Characterization of the desired product has been reported previously (Macauley et al., 2005).

Synthesis of 3,4,6-tri-O-acetyl-1,2-dideoxy-2′-propyl-β-D-glucopyranoso-[2,1-d]-Δ2′-thiazoline – 1,3,4,6-tetra-*O*-acetyl-2-deoxy-2-butyrylamido-β-D-glucopyranose (100 g, 1 eq., 0.248 mol) was added to 400 mL of dry toluene. Lawesson's reagent (60 g, 0.6 eq., 0.149 mol) was added and the reaction was stirred and heated to 80 °C for two hr in an oil bath. Typically, TLC analysis of the reaction mixture revealed that the product had completely disappeared within two hr; however, in instances where it had not, more Lawesson's reagent was added and the reaction was allowed to proceed for another hr. Following completion of the reaction, the solution was cooled to room temperature and poured on top of 1 kg of pre-washed silica in a large (15 cm diameter, 3 L volume) sintered glass filter funnel. The silica was washed with 12 L of toluene to remove Lawesson's reagent by-products. In this solvent system, the desired compound did not move through the silica. The eluent was then switched to ethyl acetate/hexanes in a 1:4 ratio. Using this solvent system, the desired product did not elute from the silica after using 12 L, whereas additional highly non-polar reaction by-products were removed by this wash. The solvent system was then switched to ethyl acetate/hexanes in a 1:1 ratio and the desired compound eluted in approximately 16 L. After vacuum evaporation of the solvent, an oil was left that was slightly yellow. Based on previous analytical samples, the faint yellow colouration of the material was due to the presence of unidentified contaminants. By NMR, the desired product accounted for > 95 % of the material but it could not be recrystallized even if it was further purified. This material was used in the next step without further purification. The yield for this step was typically in the range of 60-75% after the chromatography. Characterization of the desired product has been reported previously (Macauley et al., 2005).

Synthesis of 1,2-dideoxy-2′-propyl-β-D-glucopyranoso-[2,1-d]-Δ2′-thiazoline – 3,4,6-tri-*O*-acetyl-1,2-dideoxy-2′-propyl-β-D-glucopyranoso-[2,1-d]-Δ2′-thiazoline (50 g) was added to 400 mL of dry methanol and stirred at room temperature. Sodium methoxide was added until the solution was basic (pH > 10). The pH of the solution was monitored closely over the course of the reaction to ensure this basicity was maintained. Typically, after 30 min the reaction was judged to be complete by TLC analysis. Acetic acid diluted 1:20 into methanol was used to carefully neutralize the reaction. At this point, the product could be crystallized by the addition of ethyl acetate. Although the product appeared clean by TLC analysis, NMR spectroscopy revealed a small amount of an unidentified contaminant that was likely carried over from the starting material. Therefore, rather than immediate crystallization, the contaminant was removed by chromatography. To do so, the reaction was first concentrated by vacuum evaporation to a minimal volume (≈ 20 mL) then diluted ten-fold into ethyl acetate. Dilution had to be done slowly and carefully to avoid rapid crystallization and crashing out of the desired material. This solution was added to 1 kg of pre-washed silica and then the column was washed using ethyl acetate/methanol in a 1:20 ratio. Using this solvent system, the product was retained on the silica but the yellowish contaminant eluted. The desired product was then eluted from the column using a solvent system of ethyl acetate/methanol in a 1:5 ratio in approximately 10 L. The eluent containing the desired product was concentrated by vacuum evaporation. During concentration, the product began to crystallize after the amount of remaining solvent was less 500 mL. Once crystallization started, evaporation was stopped, the flask was placed in a refrigerator, and the material was allowed to fully crystallize over a period of several hr at 4 °C. The desired product formed fluffy white

crystals that were filtered and washed with ethyl acetate. The mother liquor was concentrated and recrystallized to increase the recovered of the product. The product was dried overnight in regular atmosphere on absorbent filter paper in a fume hood and then dried under a vacuum for two hr. The yield for this final step ranged from 50 to 75 % of isolated material following column chromatography and crystallization.

Western Blotting

Samples were separated by SDS-PAGE (10% gels), transferred to nitrocellulose membrane (Bio-Rad), blocked for one hour at room temperature (RT) with 1% bovine serum albumin (BSA) (Bioshop) in PBS containing 0.1% Tween-20 (Sigma) (PBS-T) and then subsequently probed with the appropriate primary antibody delivered in 1% BSA in PBS-T for overnight at 4 ºC. Membranes were then extensively washed with PBS-T, blocked again for 30 minutes with 1% BSA in PBS-T at RT and then probed with the appropriate HRP conjugated secondary antibody for one hr at RT delivered in 1% BSA in PBS-T. Finally, the membranes were washed extensively and then developed with SuperSignal West Pico Chemiluminesence substrate (Pierce).

Immunoprecipitation

Tissues were homogenized by manual grinding into a fine powder followed and homogenized (100 mg/ml) in cell lysis buffer (50 mM NaH_2PO_4, 150 mM NaCl, 0.5% NP-40, protease inhibitor tablet (Roche), 1 mM EDTA, and 1 mM NButGT, pH 7.4) using a tissue homogenizer (T-18 Ultra-Turrax). 100 mg of the ground tissue was dissolved in 2 ml of homogenization buffer. Insoluble cell debris was removed by centrifugation at 17,900 rcf for 20 minutes. Clear homogenates (400 μL) were added to Protein A/G – Agarose beads (40 μL) (Calbiochem) that had 5 μl of α-Sp1 pre-bound. The combined lysates and beads were rocked at 4 °C for two hours and these washed four times with lysis buffer. Immunoprecipitates were eluted by boiling in 100 μL of 1X SDS-PAGE loading buffer for five minutes. *O*-GlcNAc levels on Sp1 were detected using the *O*-GlcNAc antibody RL2 (Abcam).

Pharmacokinetic analysis of the NButGT in the blood

A male Sprague-Dawley rat six-weeks of age was given a 50 $mg{\cdot}kg^{-1}$ tail vein injection of NButGT. A various times, 200 μL of blood was collected, allowed to clot for 30 minutes at room temperature and then centrifuged at 2000 x g to isolate the serum. The serum was stored at -20 °C until further use. NButGT concentration in the serum samples was measured using an enzyme inhibition assay. First, serum obtained from the animal several hours prior to the experiment was spiked to various concentrations of NButGT. A small aliquot of this was added to an enzymatic assay consisting of 500 μM pNP-GlcNAc and 100 nM *O*-GlcNAcase in PBS (pH 7.4) and the reaction was monitored continuously in a spectrophotometer at 400 nM. Serum alone did not produce a significant rate, nor did it inhibit *O*-GlcNAcase. A K_I value was derived from a Dixon plot and this line was used as a standard curve. Serum from the animal following its injection of NButGT was then analyzed by the same enzyme assay and concentrations of the inhibitor present in each sample were derived from the standard curve. When necessary, dilutions of the serum were done in serum void of inhibitor. Values for inhibitor concentration were plotted versus time and fit to a single exponential equation using GraFit.

Determination of free fatty acid, triglyceride, insulin, and leptin levels

Prior to the hyperinsulemic-euglycemic clamp, a blood sample was taken from animals treated for eight months with NButGT and control animals. Upon isolation of the serum,

samples were stored at -20 °C under further use. Free fatty acid and triglyceride content in the plasma was determined using the appropriate kits (Sigma). Leptin levels were quantified using an ELISA (Crystal Chem). Insulin levels in the serum were quantified with an ELISA (Alpco).

Preparation of rat tissues for immunohistochemistry
Experimental and control rats were sacrificed by using CO_2, perfused transcardially with 60ml 0.1M PBS (pH 7.4) followed by 60 mL 4% (w/v) paraformaldehyde (PFA, pH 7.4). Pancreases were dissected out, post-fixed in 4% PFA for 24 hours, and then transferred to 20% (w/v) sucrose for 24 hours for cryoprotection. Pancreases were embedded with O.C.T. (optimal cutting temperature) embedding medium (Sakura Finetek USA Inc), and sectioned in the transverse plane at 30um on a cryostat. The pancreas sections were collected on *Superfrost*/Plus (Fisher Scientific) slides.

Immunohistochemistry
Tissue samples from control and chronic NButGT treated rats were processed according to a previous procedures (Shan et al., 2005). Briefly, the pancreas sections were rinsed 3 times with 0.1M PBS containing 0.3% Triton X-100 (PBS-T) for 45 minutes. After blocking with 10% NGS and 2.5% bovine serum albumin (BSA) in PBS-T for 60 minutes, sections were incubated with primary antibodies at 4 °C for 24 hours at the following dilutions: mouse anti-*O*-GlcNAc monoclonal IgM antibody at 1:500 (CTD 110.6, Covance), guinea pig anti-insulin polyclonal IgG antibody at 1:1000 (Dako). After washing 3 times with PBS-T, sections were incubated with secondary antibodies including donkey anti-mouse IgM conjugated with FITC and donkey anti-guinea pig-IgG conjugated with Cy3 (Jackson ImmunoResearch) for 90 minutes. Following a further 3 washes in PBS-T, the sections were coverslipped with Vectashield mounting medium with 4’,6-diamidino-2-phenylindole (DAPI, stains nuclei) (Vector Laboratory Inc, USA). The stained sections were examined and imaged by Leica DM4000B fluorescent microscope equipped with Leica DFC 350 FC digital camera, and images were acquired and processed by Leica LAS system.

Hematoxylin Staining
Slides containing pancreas sections were rinsed 3 times with deionized distilled water, and incubated with Harris hematoxylin (Sigma, HHS-16) for 60 seconds. They were rinsed in running tap water to remove excess stain for 3-5 minutes to “blue” the sections. Then sections were dehydrated through an ascending alcohol bath series (70%, 95%, 100%, 1 min in each), cleared in xylene 2 times for 2 min each. Slides were coverslipped with Permount (Sigma), and air dried overnight.

Analysis of Ganglioside Levels
Brains were manually grinded into a fine powder. 100 mg of frozen brain powder was homogenized by a tissue homogenizer (Ultra Turrax, Ika) in 1 ml of water. The lysates were cleared by centrifugation at 17,000 rcf for 20 min. The lipids from this extract were extracted with 3.5 ml of chloroform methanol (2:1) in a similar manner as described previously (Stubbs et al., 2009; Yowler et al., 2002). After lyophilization, the extracts were resuspended at 5 mg/mL in 2:1 chloroform methanol. Approximately 100 μg (20 μL) of extract was loaded per lane along with 1 μg mixture of GM1, GM2, and GM3 (Axxion) as standards onto a high performance TLC plate (Merck). The plate was run for one hour in a solvent system consisting of 55:45:10 chloroform/methanol/water (containing 0.2% $CaCl_2$). Following this, the plate was dried, lightly sprayed with resorcinol (80% concentrated HCl, 0.2% resorcinol, 0.25 mM $CuSO_4$), covered with a

glass plate and incubated in an oven at 100 °C for 20 minutes. For documentation, the plates were scanned on a Typhoon imager using an excitation wavelength of 523 nM and no emission filter. Densitometry was performed using ImageQuant (Molecular Dynamic).

SUPPLEMENTAL REFERENCES

Boehmelt, G., Wakeham, A., Elia, A., Sasaki, T., Plyte, S., Potter, J., Yang, Y., Tsang, E., Ruland, J., Iscove, N.N.*, et al.* (2000). Decreased UDP-GlcNAc levels abrogate proliferation control in EMeg32-deficient cells. Embo J. *19*, 5092-5104.

Cheung, W.D., and Hart, G.W. (2008). AMP-activated protein kinase and p38 MAPK activate O-GlcNAcylation of neuronal proteins during glucose deprivation. J. Biol. Chem. *283*, 13009-13020.

Cheung, W.D., Sakabe, K., Housley, M.P., Dias, W.B., and Hart, G.W. (2008). *O*-linked beta-*N*-acetylglucosaminyltransferase substrate specificity is regulated by myosin phosphatase targeting and other interacting proteins. J. Biol. Chem. *283*, 33935-33941.

Clarke, A.J., Hurtado-Guerrero, R., Pathak, S., Schuttelkopf, A.W., Borodkin, V., Shepherd, S.M., Ibrahim, A.F., and van Aalten, D.M. (2008). Structural insights into mechanism and specificity of *O*-GlcNAc transferase. Embo J. *27*, 2780-2788.

Comtesse, N., Maldener, E., and Meese, E. (2001). Identification of a nuclear variant of MGEA5, a cytoplasmic hyaluronidase and a beta-*N*-acetylglucosaminidase. Biochem. Biophys. Res. Commun. *283*, 634-640.

Dentin, R., Hedrick, S., Xie, J., Yates, J., 3rd, and Montminy, M. (2008). Hepatic glucose sensing via the CREB coactivator CRTC2. Science *319*, 1402-1405.

Fielenbach, N., and Antebi, A. (2008). C. elegans dauer formation and the molecular basis of plasticity. Genes Dev. *22*, 2149-2165.

Forsythe, M.E., Love, D.C., Lazarus, B.D., Kim, E.J., Prinz, W.A., Ashwell, G., Krause, M.W., and Hanover, J.A. (2006). Caenorhabditis elegans ortholog of a diabetes susceptibility locus: oga-1 (*O*-GlcNAcase) knockout impacts *O*-GlcNAc cycling, metabolism, and dauer. Proc. Natl. Acad. Sci. U. S. A. *103*, 11952-11957.

Fujiki, R., Chikanishi, T., Hashiba, W., Ito, H., Takada, I., Roeder, R.G., Kitagawa, H., and Kato, S. (2009). GlcNAcylation of a histone methyltransferase in retinoic-acid-induced granulopoiesis. Nature *459*, 455-459.

Gambetta, M.C., Oktaba, K., and Muller, J. (2009). Essential role of the glycosyltransferase sxc/Ogt in polycomb repression. Science *325*, 93-96.

Hanover, J.A., Forsythe, M.E., Hennessey, P.T., Brodigan, T.M., Love, D.C., Ashwell, G., and Krause, M. (2005). A Caenorhabditis elegans model of insulin resistance: altered macronutrient storage and dauer formation in an OGT-1 knockout. Proc. Natl. Acad. Sci. U. S. A. *102*, 11266-11271.

Housley, M.P., Rodgers, J.T., Udeshi, N.D., Kelly, T.J., Shabanowitz, J., Hunt, D.F., Puigserver, P., and Hart, G.W. (2008). *O*-GlcNAc regulates FoxO activation in response to glucose. J. Biol. Chem. *283*, 16283-16292.

Housley, M.P., Udeshi, N.D., Rodgers, J.T., Shabanowitz, J., Puigserver, P., Hunt, D.F., and Hart, G.W. (2009). A PGC-1alpha-*O*-GlcNAc transferase complex regulates FoxO transcription factor activity in response to glucose. J. Biol. Chem. *284*, 5148-5157.

Iyer, S.P., Akimoto, Y., and Hart, G.W. (2003). Identification and cloning of a novel family of coiled-coil domain proteins that interact with *O*-GlcNAc transferase. J. Biol. Chem. *278*, 5399-5409.

Knight, Z.A., and Shokat, K.M. (2007). Chemical genetics: where genetics and pharmacology meet. Cell *128*, 425-430.

Kreppel, L.K., Blomberg, M.A., and Hart, G.W. (1997). Dynamic glycosylation of nuclear and cytosolic proteins. Cloning and characterization of a unique *O*-GlcNAc transferase with multiple tetratricopeptide repeats. J. Biol. Chem. *272*, 9308-9315.

Kreppel, L.K., and Hart, G.W. (1999). Regulation of a cytosolic and nuclear *O*-GlcNAc transferase. Role of the tetratricopeptide repeats. J. Biol. Chem. *274*, 32015-32022.

Lubas, W.A., Frank, D.W., Krause, M., and Hanover, J.A. (1997). *O*-Linked GlcNAc transferase is a conserved nucleocytoplasmic protein containing tetratricopeptide repeats. J. Biol. Chem. *272*, 9316-9324.

Macauley, M.S., Stubbs, K.A., and Vocadlo, D.J. (2005). *O*-GlcNAcase catalyzes cleavage of thioglycosides without general acid catalysis. J. Am. Chem. Soc. *127*, 17202-17203.

Macauley, M.S., and Vocadlo, D.J. (2010). Increasing *O*-GlcNAc levels: An overview of small-molecule inhibitors of *O*-GlcNAcase. Biochim. Biophys. Acta. *1800*, 107-121.

Martinez-Fleites, C., Macauley, M.S., He, Y., Shen, D.L., Vocadlo, D.J., and Davies, G.J. (2008). Structure of an *O*-GlcNAc transferase homolog provides insight into intracellular glycosylation. Nat. Struct. Mol. Biol. *15*, 764-765.

Marz, P., Stetefeld, J., Bendfeldt, K., Nitsch, C., Reinstein, J., Shoeman, R.L., Dimitriades-Schmutz, B., Schwager, M., Leiser, D., Ozcan, S., *et al.* (2006). Ataxin-10 interacts with *O*-linked beta-*N*-acetylglucosamine transferase in the brain. J. Biol. Chem. *281*, 20263-20270.

Nimura, K., Ura, K., Shiratori, H., Ikawa, M., Okabe, M., Schwartz, R.J., and Kaneda, Y. (2009). A histone H3 lysine 36 trimethyltransferase links Nkx2-5 to Wolf-Hirschhorn syndrome. Nature *460*, 287-291.

Rossetti, L., and Giaccari, A. (1990). Relative contribution of glycogen synthesis and glycolysis to insulin-mediated glucose uptake. A dose-response euglycemic clamp study in normal and diabetic rats. J. Clin. Invest. *85*, 1785-1792.

Shan, X., Hu, J.H., Cayabyab, F.S., and Krieger, C. (2005). Increased phospho-adducin immunoreactivity in a murine model of amyotrophic lateral sclerosis. Neuroscience *134*, 833-846.

Sinclair, D.A., Syrzycka, M., Macauley, M.S., Rastgardani, T., Komljenovic, I., Vocadlo, D.J., Brock, H.W., and Honda, B.M. (2009). Drosophila *O*-GlcNAc transferase (OGT) is encoded by the Polycomb group (PcG) gene, super sex combs (sxc). Proc. Natl. Acad. Sci. U. S. A. *106*, 13427-13432.

Slawson, C., Lakshmanan, T., Knapp, S., and Hart, G.W. (2008). A mitotic GlcNAcylation/phosphorylation signaling complex alters the posttranslational state of the cytoskeletal protein vimentin. Mol. Biol. Cell. *19*, 4130-4140.

Stubbs, K.A., Macauley, M.S., and Vocadlo, D.J. (2009). A selective inhibitor Gal-PUGNAc of human lysosomal beta-hexosaminidases modulates levels of the ganglioside GM2 in neuroblastoma cells. Angew. Chem. Int. Ed. Engl. *48*, 1300-1303.

Takahashi, M., Inoue, K., Yoshida, M., Morikawa, T., Shibutani, M., and Nishikawa, A. (2009). Lack of chronic toxicity or carcinogenicity of dietary N-acetylglucosamine in F344 rats. Food Chem. Toxicol. *47*, 462-471.

Wells, L., Gao, Y., Mahoney, J.A., Vosseller, K., Chen, C., Rosen, A., and Hart, G.W. (2002). Dynamic *O*-glycosylation of nuclear and cytosolic proteins: further characterization of the nucleocytoplasmic beta-*N*-acetylglucosaminidase, *O*-GlcNAcase. J. Biol. Chem. *277*, 1755-1761.

Yang, X., Ongusaha, P.P., Miles, P.D., Havstad, J.C., Zhang, F., So, W.V., Kudlow, J.E., Michell, R.H., Olefsky, J.M., Field, S.J., *et al.* (2008). Phosphoinositide signalling links *O*-GlcNAc transferase to insulin resistance. Nature *451*, 964-969.

Yang, X., Su, K., Roos, M.D., Chang, Q., Paterson, A.J., and Kudlow, J.E. (2001). *O*-linkage of *N*-acetylglucosamine to Sp1 activation domain inhibits its transcriptional capability. Proc. Natl. Acad. Sci. U. S. A. *98*, 6611-6616.

Yowler, B.C., Kensinger, R.D., and Schengrund, C.L. (2002). Botulinum neurotoxin A activity is dependent upon the presence of specific gangliosides in neuroblastoma cells expressing synaptotagmin I. J. Biol. Chem. *277*, 32815-32819.

Zeidan, Q., Wang, Z., De Maio, A., and Hart, G.W. (2010). O-GlcNAc Cycling Enzymes Associate with the Translational Machinery and Modify Core Ribosomal Proteins. Mol Biol Cell *In Press*.
